# Use of Wearable Technology and Social Media to Improve Physical Activity and Dietary Behaviors among College Students: A 12-Week Randomized Pilot Study

**DOI:** 10.3390/ijerph16193579

**Published:** 2019-09-25

**Authors:** Zachary C. Pope, Daheia J. Barr-Anderson, Beth A. Lewis, Mark A. Pereira, Zan Gao

**Affiliations:** 1 Division of Epidemiology & Community Health, School of Public Health, University of Minnesota, 1300 S. 2nd St., Suite 300, Minneapolis, MN 55455, USA; perei004@umn.edu; 2 School of Kinesiology, University of Minnesota, 1900 University Ave. S.E., Minneapolis, MN 55455, USA; barra027@umn.edu (D.J.B.-A.); blewis@umn.edu (B.A.L.); gaoz@umn.edu (Z.G.)

**Keywords:** health behavior change, theory, physiological health, Polar M400, facebook

## Abstract

College students demonstrate poor physical activity (PA) and dietary behaviors. We evaluated the feasibility of a combined smartwatch and theoretically based, social media-delivered health education intervention versus a comparison on improving college students’ health behaviors/outcomes. Thirty-eight students (28 female; X_age_ = 21.5 ± 3.4 years) participated in this two-arm, randomized 12-week pilot trial (2017–2018). Participants were randomized into: (a) experimental: Polar M400 use and twice-weekly social cognitive theory- and self-determination theory-based Facebook-delivered health education intervention; or (b) comparison: enrollment only in separate, but content-identical, Facebook intervention. Primary outcomes pertained to intervention feasibility. Secondary outcomes included accelerometer-estimated PA, physiological/psychosocial outcomes, and dietary behaviors. Intervention adherence was high (~86%), with a retention of 92.1%. Participants implemented health education tips 1–3 times per week. We observed experimental and comparison groups to have 4.2- and 1.6-min/day increases in moderate-to-vigorous PA (MVPA), respectively, at six weeks—partially maintained at 12 weeks. In both groups, similarly decreased body weight (experimental = −0.6 kg; comparison = −0.5 kg) and increased self-efficacy, social support, and intrinsic motivation were observed pre- and post-intervention. Finally, we observed small decreases in daily caloric consumption over time (experimental = −41.0 calories; comparison = −143.3). Both interventions were feasible/of interest to college students and demonstrated initial effectiveness at improving health behaviors/outcomes. However, smartwatch provision may not result in an additional benefit.

## 1. Introduction

College students demonstrate increased overweight/obesity risk given this population’s newfound autonomy and the responsibility of balancing school, work, social, and health demands after beginning college [1,2]. College students have demonstrated insufficient levels of physical activity (PA) and high levels of sedentary behavior (SB) [3] while also engaging in poor dietary behaviors (e.g., low fruit, vegetable, and whole grain intake; high sugar-sweetened beverage [SSB] consumption) [4,5]. These behaviors have contributed to college student obesity rates of 25%–30% [2], with up to 4 kg of weight gain reported during the first two years of college attendance [5,6]. Upward weight trajectories in young adulthood are concerning and observed to be predictive of higher body mass index (BMI) in middle age [7]—contributing to the subsequent health and economic burden of overweight/obesity-related disease [8]. Given modern-day technology’s ubiquitous nature, researchers have been investigating/advocating for health behavior change interventions employing technology capable of population-level health promotion [9,10].

Smartwatch technology is currently used frequently in health behavior change interventions [11,12]. These devices allow individuals to track metrics such as steps/day which can be viewed on the device and/or an associated smartphone/internet-based application—facilitating health behavior self-regulation (i.e., tracking and modification) [13,14]. Despite these capabilities, smartwatch-based randomized trials in college students have demonstrated mixed effectiveness. For example, college students’ Misfit Flash smartwatch use and health education course participation did not improve moderate-to-vigorous PA (MVPA) or reduce SB among experimental participants versus comparison [15]. Moreover, Melton et al. [16] indicated no increase in steps/day among college students provided a Jawbone UP versus comparison participants using the MyFitnessPal smartphone application. Yet, another study observed marginally increased PA among medical students using the Fitbit versus control [17]. These mixed observations mirror smartwatch-based randomized trials in overweight/obese adults, older adults, and post-menopausal women [18,19,20].

The limitations of the preceding trials explain the mixed observations. First, most studies concentrated solely on PA, but not dietary behavior. Combined PA and dietary interventions have demonstrated greater effectiveness than interventions concentrating on either behavior exclusively [21]. Future studies must therefore consider PA and dietary health education—perhaps delivered via social media given this technology’s ability to reach large, diverse populations in a manner well-integrated into modern lifestyles [9]—particularly college students’ lifestyles [22]. Notably, PA interventions delivered to college students via social media have demonstrated the ability to increase PA levels [23]. Yet, smartwatch provision, in addition to a well-integrated social media-delivered health education intervention, may result in greater health behavior/outcome improvements as participants become more aware of their daily PA behaviors while tracking health behaviors with the smartwatch [24]. Second, future studies should consider other health outcomes (e.g., physiological, psychosocial) given the nature of overall wellness [25]. Finally, few studies employed intervention fidelity protocol. A major tenet of intervention fidelity protocol is promoting participant intervention adherence [26]. Thus, greater use of intervention fidelity protocol may improve participant adherence and, possibly, intervention effectiveness [27].

The literature has also indicated the importance of behavioral change theories for the more effective development, implementation, and analysis of health behavior change interventions [28,29]. Most prior smartwatch-based health behavior change trials have not employed any behavioral change theory. Two health behavior change theories with demonstrated success improving health behaviors are the social cognitive theory (SCT) and self-determination theory (SDT). Briefly, the SCT is predicated upon reciprocal determination between (a) individual characteristics, (b) environmental factor, and (c) behavior [30]. For instance, an individual with low PA self-efficacy (i.e., situational self-confidence) and within an unsupportive environment for PA is less likely to engage in PA behavior. Health behavior change interventions built upon the SCT have improved college students’ PA behaviors [31,32,33]. The SDT concerns the promotion of intrinsic motivation by assisting individuals in meeting three basic needs: (a) self-determination/autonomy, (b) competence, and (c) relatedness/social interaction. As these needs are met for a behavior, greater degrees of intrinsic motivation will ensue—often conceptualized along a continuum from amotivation to intrinsic motivation (Figure S1). Increased intrinsic motivation has been observed predictive of college student PA and dietary behaviors [34,35].

The objective of this 12-week pilot randomized trial was to investigate the feasibility and initial effectiveness of an intervention combining Polar M400 smartwatch use and a twice-weekly SCT- and SDT-based Facebook-delivered health education intervention on improving college students’ PA and dietary behaviors. The preceding combined intervention was compared to a group of participants enrolled only in a separate, but content-identical, Facebook-delivered health education intervention (i.e., comparison). Given the study’s pilot nature, our primary outcomes included intervention interest, use/acceptability, adherence, and retention, as recommended by the National Institutes of Health’s National Center for Complementary and Integrative Health (NCCIH) [36] and others [37] for the reporting of pilot trials. Our secondary outcomes included changes to PA, SB, physiological, SCT- and SDT-related psychosocial, and dietary outcomes from baseline to 12 weeks. Relative to comparison participants, we hypothesized that experimental participants receiving the combined intervention would demonstrate greater improvements in these secondary outcomes during the intervention. Our observations build upon the limitations of past trials and add to the literature given: (a) the concurrent concentration on PA, SB, and dietary behaviors; (b) the use of the SCT and SDT as theoretical frameworks in the intervention design; (c) the use and implementation of SCT- and SDT-based health education delivered via social media; (d) the inclusion of physiological and psychosocial outcomes in addition to behavioral outcomes related to PA, SB, and diet; and (e) the inclusion of routine intervention fidelity procedures employed to promote intervention adherence. Researchers and health professionals on college campuses may modify the methods employed in the current trial to increase intervention effectiveness based upon our observations.

## 2. Materials and Methods

Consolidated standards of reporting trials (CONSORT) guidelines [38] were used when drafting this manuscript. This study was approved by the University of Minnesota Institutional Review Board (Study #: STUDY00000386) in June 2017, with the study also registered at ClinicalTrials.gov (Study #: NCT03253406).

### 2.1. Participants

Thirty-eight college students from a large metropolitan Midwest University participated in Fall 2017/Spring 2018. We recruited via flyer/email communication and in-person recruitment presentations. Inclusion criteria were: (a) 18–35 years old; (b) BMI ≥18.5 kg/m^2^; (c) PA levels below national recommendations [39] over the last month—verified via scripted screening interviews; (d) currently eating less than the recommended daily amount of fruit and vegetables, respectively [40]—verified using food frequency questionnaire [41]; (e) no self-reported diagnosed physical/mental disability; (f) completed physical activity readiness questionnaire; and (g) willing to be randomized. University of Minnesota Institutional Review Board (IRB) approval and participant consent were obtained prior to recruitment/data collection. All participant procedures were performed in accordance with the ethical standards of the Institution and/or national research committee and with the 1964 Helsinki Declaration and its later amendments or comparable ethical standards [42].

### 2.2. Study Design

We used a 12-week two-arm randomized pilot trial design, with participants randomized into (a) the experimental group: provided a Polar M400 smartwatch to track PA duration and steps/day and included in a Facebook group wherein SCT- and SDT-based PA and nutritious eating health education tips were provided twice weekly, or (b) a comparison group: included only in separate, but content-identical, Facebook group, with no smartwatch provided.

### 2.3. Data Collection Instruments

#### 2.3.1. Primary Outcome

*Intervention Interest, Use/Acceptability, Adherence, and Retention.* We operationalized intervention interest as the number of college students contacting us with study interest over our cumulative recruitment duration of six weeks. Intervention use/acceptability was evaluated in a few ways. Briefly, we surveyed both groups regarding Facebook-delivered health education tip helpfulness (1—very unhelpful; 7—very helpful) and tip implementation frequency. The experimental group received additional questions regarding the Polar M400’s usefulness for PA tracking/modification (*1—*not very useful; 7—very useful) and ease-of-use (1—very difficult; 7—very easy), with positive and negative device features reported on two open-ended, qualitative questions. We assessed both groups’ adherence using the “Like” and “Seen By” functions on Facebook. Specifically, we asked participants to “Like” any health education tip they read. If a participant “Liked” and was registered as having seen the post (“Seen By” function), that individual was given one “adherence point”. Twenty-four points were available (two weekly posts × 12 weeks), with percentage adherence calculated by dividing the # of points received by 24 and multiplying by 100%. Finally, we calculated intervention retention as: (# of participants randomized and beginning the trial/# of participants who finished the trial) * 100%.

#### 2.3.2. Secondary Outcomes

Physical Activity was assessed using ActiGraph Link accelerometers worn on the wrist given a Polar M400 validation sub-study completed during this trial (not reported). As no previously validated wrist-based cut points existed for data analysis, we used the Link’s raw data to complete a minute-by-minute stepping rate analysis [43,44]. The following cut points—in steps/minute—were used to assess time in different PA intensities: SB: 0–19; LPA: 20–99; and MVPA: ≥100. Participants wore the Link for seven days at baseline, six weeks, and 12 weeks, with at least 12 h/day of validated wear time from two weekdays and one weekend day used for analyses [45,46]. Following data validation, we exported/analyzed raw Link data within Microsoft Excel (Microsoft Inc.; Redmond, WA, USA) where “COUNTIFS” and “AVERAGE” functions were employed to determine mean SB, LPA, and MVPA using these cut points.

Cardiorespiratory Fitness was valuated with the YMCA 3-Minute Step Test [47] at baseline and 12 weeks. Participants’ stepping cadence was dictated by a metronome set at 96 beats/minute, with the test completed on a 12-inch riser. Participants’ heart rate was assessed immediately following the test via radial artery palpation.

With respect to height, weight, and body composition, height was measured to the nearest 0.5 cm using a Seca stadiometer (Seca; Hamburg, Germany), with weight measured to the nearest 0.1 kg using a Tanita BC-558 IRONMAN^®^ scale (Tanita; Tokyo, Japan). Participants wore lightweight athletic clothing. This scale also conducted bioelectrical impedance assessments—a valid field measure of young adults’ body composition [48]. Measurements occurred at baseline and 12 weeks.

Social Cognitive Theory-Related Psychosocial Constructs. Psychometrically validated questionnaires assessed SCT-related psychosocial constructs at baseline and 12 weeks. A six-item questionnaire [49] examined participants’ self-efficacy in overcoming certain barriers (e.g., “…exercise when I am tired”) (1—not confident at all; 5—extremely confident). Social support was evaluated via a five-item questionnaire [49] with participants rating how often others provide health behavior-related support/encouragement (1—almost never to 5—almost always). A modified five-item questionnaire [50,51] assessed participants’ enjoyment of health-related behaviors. Specifically, participants rated agreement with statements like “I have more fun engaging in physical activity than doing other things” (1—disagree to 3—agree). A 14-item questionnaire examined participants’ perceived health behavior barriers by asking participants to rate agreement between perceived barriers and hypothetical barriers (1—strongly disagree to 4—strongly agree) [52]. Finally, participants’ outcome expectancy was assessed via a modified 12-item dichotomous (1—Yes; 0—No) questionnaire [53,54]. The internal consistency (i.e., Cronbach’s alpha) for these measures were acceptable to good in our sample (self-efficacy: 0.73; social support: 0.81; enjoyment: 0.70; barriers: 0.78; and outcome expectancy: 0.72) [55].

Self-Determination Theory-Related Intrinsic Motivation. Evaluated using interest/enjoyment subscale of the validated Intrinsic Motivation Inventory [56] at baseline and 12 weeks. This seven-item questionnaire required participants to determine how true statements like, “I enjoyed this activity very much”, were to them (1—not at all true; 7—very true). Cronbach’s alpha indicated excellent internal consistency for this measure (0.95) in our sample.

Dietary Behaviors were examined using the National Cancer Institute’s Automated Self-Administered 24-h (ASA24) food recall [57,58]. We provided participants unique ASA24 login information and administered the ASA24 three times on non-consecutive random dates (two weekdays; one weekend day) at baseline, six weeks, and 12 weeks (nine total recalls). Multiple food recalls are needed for the accurate estimation of habitual intake and comparison to dietary recommendations [59,60]. Outcomes were daily intakes of calories, fruits and vegetables (cups), whole grains (ounce equivalents), and SSB (calories). SSB were defined as any of the following non-alcoholic beverages: sports drinks, fruit drinks/punches (those not 100% juice), soda, low-calorie drinks, sweetened tea/coffee, and other sweetened beverages (e.g., flavored milk) [61,62].

### 2.4. Procedures

Interested students first came to the University laboratory for baseline screening. Participants meeting inclusion criteria completed baseline testing, with SCT and SDT psychosocial construct questionnaires administered first and height, weight, body fat percentage, and cardiorespiratory fitness evaluated thereafter. We then provided participants with an ActiGraph Link to wear for the baseline seven-day testing period during all waking hours. Participants were also provided a wear log to document wear times, with this document containing wear instructions to reinforce adherence. Finally, each participant completed an ASA24 food recall tutorial involving the input of an actual meal they ate the previous day. Participants were encouraged to contact Author #1 with questions and were contacted every three weeks during the study with standardized emails encouraging continued adherence (i.e., intervention fidelity protocol).

Following seven-day baseline testing, participants were informed of group allocation—determined using a random numbers table and a 1:1 allocation ratio by Author #1. Participants randomized into the experimental group received the Polar M400 smartwatch, associated manuals/accessories, and a detailed tutorial of device use. Experimental participants were subsequently placed within the Facebook group wherein SCT- and SDT-based health education tips were provided twice weekly. Briefly, on Mondays, participants were asked to read a PA-related health education tip and to read a nutrition-related health education tip on Thursdays. For example, to increase PA-related self-efficacy and self-determination while simultaneously decreasing PA-related barriers, participants were informed that three daily 10-min PA bouts are enough to meet national PA recommendations. Similar tips were developed for nutritious eating behaviors, with all tips located within Appendix A. Participants randomized into the comparison group were placed within a separate, but content-identical, Facebook group and asked to discontinue smartwatch use for the study’s duration. Participants were paid $30 for successful study completion (i.e., all data collection sessions completed and study device[s] returned).

### 2.5. Statistical Analyses

SPSS 25.0 (IBM Inc., Armonk, NY, USA) was used for all statistical analyses. We used histograms and Shapiro-Wilks statistics to examine for outliers. Chi-square and independent t-tests were then used evaluate baseline group differences in categorical and continuous variables, respectively. We reported primary outcomes (e.g., metrics related to intervention feasibility) descriptively. Recommendations from the NCCIH [36] and others [37] for the reporting of pilot trials dissuade the use of inferential statistics when reporting results related to hypothesis testing. We have therefore also reported our secondary outcomes descriptively. As a supplement, we also evaluated each participant’s changes in secondary outcomes as percent change from baseline: ([post-value—baseline value]/[baseline value] * 100%). Mean percent change for each secondary outcome by intervention arm was then reported and better allowed for the interpretation of the variability in our pilot trial’s observations for these outcomes.

## 3. Results

### 3.1. Participant Flow

**Figure 1:**
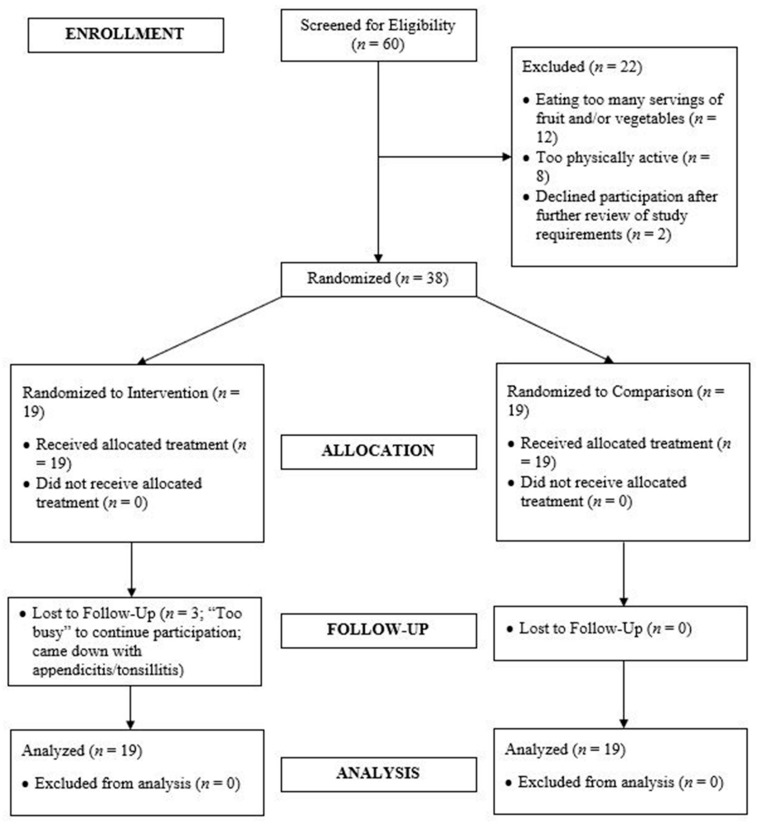
Consolidated Standards of Reporting Trials (CONSORT) Participant Flow Diagram.

Figure 1 presents the CONSORT participant flow diagram. Sixty college students were screened for study participation. Forty college students were deemed eligible; however, two participants withdrew their desire to participate after further reviewing study requirements. Thus, 38 participants completed baseline testing and randomization. Retention was 84.2% in the experimental group and 100% in the comparison group. Three experimental group participants dropped out for reasons unrelated to the study—two participants just prior to six-week testing and one participant just before 12-week testing. Dropouts baseline data was not significantly different than completers—thus all data are included in these analyses, congruent with CONSORT [38].

### 3.2. Primary Outcome

#### Intervention Interest, Use/Acceptability, Adherence, and Retention

We received inquiries regarding study interest from 126 college students over six weeks of recruitment. Groups most often reported the health education tips as “helpful”, with most participants implementing the tips one to three times weekly. The experimental group found the Polar M400 “somewhat helpful” to “helpful” in assisting to increase PA—rating the device’s ease-of-use as “somewhat easy”. Experimental participants’ comments on the Polar M400’s positive/negative features are included in Table 1. We observed high intervention adherence (experimental: 89.8 ± 21.8%; comparison: 84.4 ± 22.3%) and, as mentioned, intervention retention was high. All participants recommended their respective intervention for future implementation.

### 3.3. Secondary Outcomes

Baseline comparisons are included in Table 2. Only one baseline group difference was observed as the experimental group reported higher whole grain intake versus comparison, *t* = 2.3, *p* = 0.03.

#### 3.3.1. Physical Activity

All PA descriptive statistics by group over time are provided in Table 3. Both groups increased MVPA/day from baseline to six weeks (experimental: 4.2 min; comparison: 1.6 min). Although both groups had slight MVPA decreases from the sixth to 12th weeks, MVPA at 12 weeks was still higher than at baseline—yet, percent change values varied widely within each group. No other notable changes were observed.

#### 3.3.2. Physiological Outcomes

Table 4 provides descriptive statistics for physiological outcomes by group over time. Body fat percentage increased by 2.2% and 0.3% in the experimental and comparison groups, respectively, over time. Interestingly, slightly decreased weight was observed among both groups during the intervention (experimental: −0.6 kg; comparison: −0.5 kg), with the percent change larger in the comparison vs. experimental group (−0.5% vs. −0.2%, respectively). Finally, improved cardiorespiratory fitness was seen for the comparison group (3.3-beat/minute decrease; percent change: −2.0%) but not the experimental group (1.8-beat/minute increase; percent change: +2.6%).

#### 3.3.3. Psychosocial Outcomes

Descriptive statistics for all psychosocial outcomes by group over time are presented in Table 4. The most notable changes over time were observed for self-efficacy (experimental: +33.7%; comparison: +28.7%), social support (experimental: +36.5%; comparison: +19.0%), and intrinsic motivation (experimental: +13.7%; comparison: +12.3%). While changes in enjoyment, outcome expectancy, and barriers were smaller in magnitude, these changes were similar for both groups and in the expected directions.

#### 3.3.4. Dietary Outcomes

Table 3 contains descriptive statistics by group over time for dietary outcomes. Decreased daily caloric intake was observed for both groups (experimental: −41.0 calories, percent change: −0.9%; comparison: −143.3 calories, percent change: −4.6%). Further, the comparison group had a slightly increased vegetable consumption at six weeks versus baseline (+0.4 cups, percent change: 54.5%), but this trend was not observed at 12 weeks. The experimental group demonstrated a small decrease in vegetable consumption at six and 12 weeks (−0.2 cups for both time points), but percent change values indicated these values varied considerably. No other discernable trends were seen.

## 4. Discussion

Observations suggested that while both study interventions were feasible/of interest to college students and demonstrated initial effectiveness, the combination of the Polar M400 smartwatch and SCT- and SDT-based, Facebook-delivered health education intervention did not offer a marked advantage over a standalone SCT- and SDT-based, Facebook-delivered health education intervention. Similar between-group observations may be partially attributed to the Polar M400 smartwatch. As indicated (see Table 1), experimental participants were often frustrated with the Polar M400’s syncing capabilities, its less intuitive design versus other smartwatches, and the bigger watch size. Anecdotally, some participants stated this decreased their desire to pay attention to the watch’s data and, at times, the watch size forced them to remove the device during certain activities—possibly decreasing awareness of PA self-regulation. These observations have implications for future trials as they suggest that a smartwatch that is more mainstream, intuitive, and smaller may be needed when seeking to enhance PA awareness and self-regulation among college students when using this type of wearable technology. Below, we discuss changes in health behaviors/outcomes in relation to our hypothesis and the implications these observations have for the development and implementation of future trials.

Small increases were observed in both groups’ MVPA, with a slightly greater increase in the experimental group—particularly at six weeks—versus comparison. This observation was partially congruent with our hypothesis. Compared to previous smartwatch-based randomized trials among college students [15,16] and other populations [18,19], the current investigation’s MVPA observations are similar or marginally better despite the small magnitude of increase. Leisure-time PA has a well-known positive relationship with health [63]. Thus, the experimental group’s MVPA increase at six weeks may be practically significant as it contributed to nearly 30 min/week more PA than observed at baseline. Further, while college students’ LPA and SB durations did not change markedly, the approximate 168 and 547 min/day of LPA and SB observed are similar to durations previous smartwatch-based randomized trials [15] and epidemiological studies in this population [3,64]. College students’ high LPA durations have been suggested reflective of college students’ active transportation to and from residencies on/near campus and between classes [65,66], while high SB durations may be indicative of time spent studying, attending classes, and/or watching TV/computer use [3,64]. High SB durations are particularly disconcerting given the increasing evidence of SB’s deleterious health effects [67]. Based upon our observations, we suggest that future larger trials consider investigating ways to ensure the long-term maintenance of MVPA improvements while simultaneously working to decrease SB by more greatly leveraging social media—regardless of whether participants are provided a smartwatch to track PA and SB. For instance, using Facebook, health professionals might consider friendly weekly MVPA and SB competitions between participants which also challenge participants to implement the PA-related health education tip for that week.

Greater physiological improvements among experimental versus comparison participants were not observed—unsupportive of our hypothesis. Yet, both groups’ slightly decreased weight during the intervention is promising given the weight gain typically observed among students during their college years [5,68] and the notable positive correlations between college student weight trajectories and middle-age BMI [7]. Although both groups had slightly increased body fat percentage, this increase is within the percentage error observed for bioelectrical impedance during interventions [69] and may reflect body water level/electrolyte balance more than actual body composition changes [70]. As for cardiorespiratory fitness, the comparison group’s minor improvements and the experimental group’s small decreases might be attributable to regression to the mean as the experimental group began the study with better cardiorespiratory fitness (see Table 2 and Table 4). Regardless, future trials might consider posting workout programs to Facebook that college students can implement at their discretion to improve body weight, body fat percentage, and cardiorespiratory fitness improvements given the widespread health benefits [70,71].

Psychosocial outcome improvements were not aligned with the study’s hypothesis but encouraging nonetheless given both groups demonstrated improvements in self-efficacy, social support, and intrinsic motivation during the intervention. Studies have suggested that self-efficacy predicts college student PA participation and/or nutritious eating behaviors [31,32,72], with social support from friends and other significant figures observed most important in promoting this population’s participation in these health behaviors [33,73]. Indeed, we sought to convert negative efficacy expectations regarding health behavior participation to more positive efficacy expectations [30] using health education tips delivered via a technology-based platform wherein social support could be provided—providing a possible explanation for improvements in these two constructs. Intrinsic motivation is also associated with greater willingness to persist in proper PA and dietary behaviors among college students [34,35]. The improved intrinsic motivation we observed may be partially attributable to increased self-efficacy and social support—a fact suggested in prior research [33]. Briefly, when referencing the three basic needs within the SDT, it is plausible that: (a) participants’ increased self-efficacy may have led to greater self-determination/autonomy for proper health behavior participation; (b) participants felt greater relatedness to other participants within their respective Facebook groups also trying to improve their health—congruent with increased social support observations; and (c) participants perceived increased competence regarding health behavior participation resulting from the reading/implementation of health education tips. Future trials will have to further investigate these hypothesized interrelationships in a bigger sample.

Outcome expectancy has also been observed as a significant predictor of whether college students meet PA guidelines [74]. Both groups had high outcome expectancy scores at baseline (combined mean: 9.7; scale range: 0–12 with higher scores better). Therefore, a ceiling effect might have limited any remarkable improvement in this outcome. A similar explanation might also be posited for why little change was seen for perceived barriers (combined mean: 28.6; scale range: 14–56 with lower scores better). College students have cited multiple barriers to health behavior participation (e.g., lack of time, inconvenient recreation center hours, higher cost/longer time needed to cook healthy, etc.) [75,76]. While our barrier-related health education tips built upon barriers cited in past literature, it is possible that the study’s tips were too “generic”. These observations have implications for the design of future trials. Specifically, we believe conducting focus groups with college students to discern what important outcomes and perceived barriers are most relevant to them is warranted. This information could be used to personalize strategies for improving health-related outcome expectancy and perceived barriers while possibly increasing college student enjoyment of personally relevant health behavior participation.

Combined PA and dietary interventions have stronger effects on health outcomes versus exclusively PA-focused interventions [21,77]. Unfortunately, we did not observe either group to have improved dietary behaviors over time. While both groups had decreased caloric consumption from baseline to 12 weeks, these were modest decreases, with the small sample size precluding stating whether this is an actual trend. Further, the fruit, vegetable, and whole grain consumption levels observed were all lower than national recommendations at all time points, with no discernible trends over time. Low consumption of fruit, vegetables, and whole grains have been associated with type 2 diabetes, heart disease, and some cancers [40]. While previous web-based nutritional interventions among college students have resulted in improved dietary behaviors [33,78], these nutritional interventions were more in-depth than the current study. Specifically, given dietary behavior complexity versus PA behaviors, two weekly posts (only one of which was nutrition-related) were likely not enough to influence dietary behaviors—particularly important given the marked effect the college-built environment can have on college student dietary (and PA) behaviors [5]. Thus, future trials might consider more intensive approaches to promoting proper dietary behaviors—perhaps providing weekly healthy low-cost/easily-prepared recipes to college students using Facebook’s document posting features.

Study strengths included: (a) randomized design; (b) theory-informed intervention development, implementation, and analysis; (c) low-burden and well-integrated methodology combining two technologies (smartwatches, social media) used frequently by college students; (d) concurrent concentration on PA and dietary behavior health education; (e) high retention rate; and (f) routine implementation of intervention fidelity procedures. However, the following limitations should also be noted. First, no true control group was present which limited the study’s ability to detect the additive effect of a Facebook-delivered health education intervention exclusively versus the pairing of this intervention with smartwatch use. Future larger trials may employ a delayed-intervention control. Second, academic year breaks (i.e., Thanksgiving, Christmas, and Spring Breaks) and midterms/finals may have biased PA and dietary behaviors. Indeed, the six- and 12-week assessments often coincided with these breaks/course exam periods which could have decreased PA participation and promoted poorer dietary habits. If possible, future trials’ recruitment and intervention implementation strategies should avoid these time periods. Third, while the SCT and SDT factored heavily into this health education intervention’s development/implementation, greater use of behavioral techniques (e.g., goal setting, structured feedback) may be implemented using the novel features of Facebook groups (e.g., quizzes or polls). Finally, the study included a small homogenous sample from a single University—limiting generalizability. A multi-site trial would work well in future studies to increase sample size/diversity—thus increasing generalizability.

## 5. Conclusions

Study observations indicated both interventions within the current study to be feasible and of interest to college students. Given the call for health behavior change interventions which are low-cost, low-burden, and have wider reach among diverse populations [9] this study suggested that perhaps a standalone theoretically-based, social media-delivered health education intervention may be sufficient for scaling and the application for health promotion on a college campus—without the need for smartwatch use. However, our observations suggested that more attention needs to be paid to more in-depth implementation of behavior change strategies and use of the unique features of social media platforms like Facebook. Researchers and health professionals may consider the following in future trials among college students based upon our results: (a) posting Facebook quizzes or polls to assess participant’s comprehension of the health education tips provided; (b) using Facebook’s document posting features to provide workout programs and easy-to-make recipes; and (c) requesting that participants post their fitness goals within the group so that participants may encourage one another toward these goals.

## Figures and Tables

**Table 1:** Experimental Participants’ Comments Regarding Polar M400 Features.

	Comments
**Positive Features**	“Exercise Bar” on main screen that “filled up” as participant completed progressively more PA toward daily goal. The physical inactivity reminder which made smartwatch vibrate after 45 min of inactivity. The “Diary” function of the smartwatch that allowed for the participant to review all PA completed over the last 30 days. Capability of smartwatch to track multiple PA modalities.
**Negative Features**	Poor smartwatch to smartphone syncing. Size of the watch was often “too big”. Did not possess built in heart rate monitor. Global positioning system sometimes poor for outdoor activities.

Note: At least five participants had to provide approximately the same comment prior to inclusion of a comment in this table.

**Table 2:** Baseline Group Comparisons.

	Experimental (*n* = 19)	Comparison (*n* = 19)	*p*-Value
***Demographic and Anthropometric Variables***			
**Sex**			
**Female**	15	13	0.71
**Male**	4	6	
**Race/Ethnicity**			
**Non-Hispanic White**	16	11	0.20
**Asian**	3	8	
**Age [years]**	21.2 (4.0)	21.8 (2.8)	0.58
**Height [cm]**	171.5 (3.9)	170.0 (8.2)	0.48
**Weight [kg]**	73.4 (11.3)	69.0 (15.8)	0.33
**Body Mass Index**	24.9 (3.3)	23.8 (4.6)	0.39
***Physiological Variables***			
**Body Fat Percentage**	27.7 (8.0)	26.4 (7.0)	0.61
**Cardiorespiratory Fitness [BPM]**	104.2 (19.1)	112.5 (22.7)	0.23
***Physical Activity Variables***			
**MVPA/Day [min]**	6.1 (7.0)	5.8 (5.9)	0.90
**LPA/Day [min]**	180.7 (30.7)	161.6 (50.8)	0.17
**SB/Day [min]**	534.7 (30.3)	553.6 (50.3)	0.18
***Psychosocial Variables***			
**Self-Efficacy**	2.3 (0.4)	2.2 (0.8)	0.78
**Social Support**	2.1 (0.5)	2.2 (0.9)	0.69
**Enjoyment**	2.4 (0.4)	2.3 (0.5)	0.57
**Barriers**	27.2 (5.0)	30.0 (5.7)	0.11
**Outcome Expectancy**	9.9 (1.8)	9.5 (2.3)	0.53
**Intrinsic Motivation**	4.5 (1.0)	4.5 (1.3)	0.89
***Dietary Variables***			
**Daily Caloric Consumption [cals]**	1986 (461.9)	1953 (526.5)	0.84
**Daily Fruit Intake [cups]**	0.8 (0.5)	0.8 (0.7)	0.73
**Daily Vegetable Intake [cups]**	1.5 (0.7)	1.3 (0.7)	0.48
**Daily Whole Grain Intake [oz. equivalents]**	1.2 (0.9)	0.6 (0.7)	0.03
**Daily Sugar-Sweetened Beverage Kcalories**	110.1 (32.5)	128.0 (42.9)	0.48

Note. All categorical variable values are frequency; All continuous variable values are mean (standard deviation); BPM: beats-per-minute; MVPA: moderate-to-vigorous physical activity; LPA: light physical activity; SB: sedentary behavior; cals: calories; min: minutes.

**Table 3:** Physical Activity and Dietary Outcomes by Group at Baseline, 6 Weeks, and 12 Weeks.

	Group	Baseline	6 Weeks	% Change at 6 Weeks	12 Weeks	% Change at 12 Weeks
***Physical Activity Outcomes***						
**MVPA/Day [min]**	*Experimental*	6.1 (7.0)	10.3 (6.5)	173.1 (225.2)	8.1 (5.6)	110.7 (194.8)
	*Comparison*	5.8 (5.9)	7.4 (5.3)	134.4 (212.2)	6.7 (6.1)	44.6 (124.5)
**LPA/Day [min]**	*Experimental*	180.7 (30.7)	162.4 (30.3)	−8.1 (21.0)	164.2 (43.1)	−6.9 (25.4)
	*Comparison*	161.6 (50.8)	166.1 (63.7)	6.1 (32.8)	175.5 (51.2)	16.3 (42.8)
**SB/Day [min]**	*Experimental*	534.7 (30.3)	549.0 (29.2)	2.9 (7.4)	548.7 (44.6)	2.9 (10.0)
	*Comparison*	553.6 (50.3)	559.9 (52.4)	1.5 (9.5)	538.9 (50.3)	−2.1 (10.6)
***Dietary Outcomes***						
**Daily Kcaloric Consumption [cals]**	*Experimental*	1986.1 (461.9)	1971.7 (553.4)	−0.1 (21.4)	1945.1 (569.6)	−0.9 (23.7)
	*Comparison*	1953.4 (526.5)	1935.3 (478.0)	−0.9 (23.6)	1810.1 (512.6)	−4.6 (22.2)
**Daily Fruit Intake [cups]**	*Experimental*	0.8 (0.5)	0.8 (0.5)	31.6 (130.6)	1.0 (0.9)	36.7 (157.5)
	*Comparison*	0.8 (0.7)	0.6 (1.6)	−7.3 (99.6)	0.6 (0.7)	−7.0 (97.0)
**Daily Vegetable Intake**	*Experimental*	1.5 (0.7)	1.3 (0.7)	20.1 (118.9)	1.3 (0.5)	14.5 (22.0)
	*Comparison*	1.3 (0.7)	1.7 (1.0)	64.0 (104.7)	1.0 (0.6)	−0.7 (85.9)
**Daily Whole Grain Intake [oz. equivalents]**	*Experimental*	1.2 (0.9)	1.1 (0.8)	29.4 (132.6)	1.1 (0.9)	9.8 (89.6)
	*Comparison*	0.6 (0.7)	1.0 (0.9)	110.0 (189.0)	0.8 (1.0)	93.0 (188.0)
**Daily SSB Consumption Calories**	*Experimental*	110.1 (32.5)	279.6 (137.7)	179.9 (166.2)	147.8 (99.3)	47.1 (109.0)
	*Comparison*	128.0 (42.9)	136.8 (115.3)	1.7 (60.2)	110.8 (47.8)	−7.5 (49.1)

Note. All values are mean (standard deviation); MVPA: moderate-to-vigorous physical activity; LPA: light physical activity; SB sedentary behavior; min: minutes; cals: calories; oz. equivalents: ounce equivalents; SSB: sugar-sweetened beverages.

**Table 4:** Physiological and Psychosocial Outcomes by Group at Baseline and 12 Weeks.

	Group	Baseline	12 Weeks	% Change at 12 Weeks
***Physiological Outcomes***				
**Weight [kg]**	*Experimental*	73.4 (11.3)	72.9 (9.0)	−0.2 (5.7)
	*Comparison*	69.0 (15.8)	68.5 (14.7)	−0.5 (2.5)
**Body Fat Percentage**	*Experimental*	27.7 (8.0)	29.8 (6.4)	11.1 (17.5)
	*Comparison*	26.4 (7.0)	26.7 (7.2)	1.7 (8.0)
**Cardiorespiratory Fitness [BPM]**	*Experimental*	104.2 (19.1)	106.0 (16.9)	2.6 (9.2)
	*Comparison*	112.5 (22.7)	109.2 (20.6)	−2.0 (12.1)
***Psychosocial Outcomes***				
**Self-Efficacy**	*Experimental*	2.3 (0.4)	3.0 (0.5)	33.7 (29.3)
	*Comparison*	2.2 (0.8)	2.7 (0.9)	28.7 (63.6)
**Social Support**	*Experimental*	2.1 (0.5)	2.8 (0.7)	36.5 (47.3)
	*Comparison*	2.2 (0.9)	2.5 (1.1)	19.0 (54.6)
**Enjoyment**	*Experimental*	2.4 (0.4)	2.5 (0.3)	9.3 (24.0)
	*Comparison*	2.3 (0.6)	2.4 (0.5)	5.7 (16.3)
**Barriers**	*Experimental*	27.2 (5.0)	26.1 (4.0)	−1.7 (22.1)
	*Comparison*	30.0 (5.7)	28.0 (5.0)	−4.5 (21.0)
**Outcome Expectancy**	*Experimental*	9.9 (1.8)	10.5 (1.3)	8.3 (16.0)
	*Comparison*	9.5 (2.3)	9.8 (2.1)	7.7 (29.5)
**Intrinsic Motivation**	*Experimental*	4.5 (1.0)	5.0 (1.1)	13.7 (16.7)
	*Comparison*	4.5 (1.3)	5.0 (1.4)	12.3 (24.6)

Note. All values are mean (standard deviation). kg: kilogram; BPM: beats per minute.

## Supplementary Materials

The following are available online at https://www.mdpi.com/1660-4601/16/19/3579/s1, Figure S1: Self-Determined Motivation Continuum.

## Appendix A

**Twice-Weekly Health Education Tips**

The following tips will be provided twice a week on Monday and Thursday for 12 weeks to all study participants. Specifically, the intervention group and the comparison group will have their own Facebook groups through which these health tips will be provided. Notably, each week includes one physical activity-related posting (Monday) and one nutrition-related posting (Thursday), with Social Cognitive Theory- and Self-Determination Theory-Related health determinants targeted denoted after each tip in ***bold italics***.

**Week One**

***Physical Activity Tip:***

Did you know the American College of Sports Medicine states that the recommended 30 min a day of physical activity can be accumulated in 3 ten-minute bouts?! If you have an extra ten minutes, try going for a brisk walk or bike ride. Indeed, a quick physical activity break following a meal or at points of the day where you begin to feel tired can really go a long way! ***Social cognitive belief(s) targeted: Promoting Self-Efficacy, Decreasing Barriers; Self-determination theory constructs potentially influenced: Self-Determination/Autonomy, Competence.***

***Nutrition Tip:***

Water is vital. Although recommendations put forth by numerous health organizations such as the Centers for Disease Control and Prevention and the American College of Sports Medicine state 6–8 cups a day is needed, this does not always hold true for all individuals. Therefore, if you are not confident in your ability to drink enough water, buy a good water bottle that can be used at the gym and during the day and drink each time you feel thirsty. Why is this important? Well, drinking water consistently throughout the day can help decrease your appetite and improve digestion while also replacing unnecessary excess kcalories from beverages such as soda and fruit juices that often contain added sugars. Given the preceding benefits, drinking more water may aid in improving health and even help you lose weight! ***Social cognitive belief(s) targeted: Promoting Self-Efficacy, Improving Outcome Expectancy; Self-determination theory constructs potentially influenced: Competence.***

**Week Two**

***Physical Activity Tip:***

Social support is crucial to continued physical activity engagement. Thus, try to find a good friend willing to go “sweat it out” with you two or three times per week. Although your friend may not be able to join you for every workout, s/he may be able to provide company on the days where you are lacking the motivation to get to the gym. ***Social cognitive belief(s) targeted: Enhancing Social Support; Self-determination theory constructs potentially influenced: Relatedness/Social Interaction.***

***Nutrition Tip:***

Small changes in what you eat and drink daily will enhance the likelihood of sticking to your nutritional goals as opposed to wide, sweeping changes all at once. Indeed, you need to explore small dietary changes which best conform to your taste preferences and daily schedule while also promoting improved health. For instance, if you are busy with school or work and find you tend to purchase an unhealthy snack from a vending machine, perhaps consider a healthy snack replacement strategy where you pack a small plastic bag of carrots, cucumbers, and whole grain crackers. Not only will this alternative likely be lower in kcalories, but the foods will also contain more of the nutrients your body needs for good health. ***Social cognitive belief(s) targeted: Promoting Self-Efficacy, Decreasing Barriers; Self-determination theory constructs potentially influenced: Self-Determination/Autonomy, Competence.***

**Week Three**

***Physical Activity Tip:***

School work or your job stressing you out? Lucky for you, physical activity releases stress-reducing hormones such as endorphins into the bloodstream even during short 10-min bouts of exercise. All the more reason to set aside a little time each day to be physically active! ***Social-cognitive belief(s) targeted: Improving Outcome Expectancy; Self-determination theory constructs potentially influenced: Competence.***

***Nutrition Tip:***

Desire to introduce more fruits and vegetables into your diet but worried about these fruits and vegetables going bad before you have had a chance to eat them? Consider a trip down the freezer isle at your local grocery store. Specifically, look at the frozen fruit and vegetable options available to you! Indeed, most fruits and vegetables are available as frozen options and are every bit as nutritious as their non-frozen counterparts—and sometimes cheaper as well. Just remember to look for frozen fruit and vegetable options which do not have added sauces (e.g., gravy) or extra butter/cream and salt. ***Social cognitive belief(s) targeted: Promoting Self-Efficacy, Decreasing Barriers; Self-determination theory constructs potentially influenced: Self-Determination/Autonomy, Competence.***

**Week Four**

***Physical Activity Tip:***

Worried about your motivation to exercise in the morning or that you will forget your exercise clothes as you head out the door? Place your workout clothes/shoes in front of the door you exit each morning. In this manner, you will have to move the clothes prior to opening the door, acting as a reminder to be a little more physically active during the day or to not skip the gym in the evening. ***Social cognitive belief(s) targeted: Promoting Self-Efficacy, Decreasing Barriers; Self-determination theory constructs potentially influenced: Self-Determination/Autonomy.***

***Nutrition Tip:***

Protein is important for numerous processes in the body. Not only is protein important for growth/repair of bodily tissues, but is instrumental in the proper functioning of hormones and enzymes as well. Foods like chicken, seafood, beans, eggs, and soy products are great lean sources of protein which can be introduced into the diet as part of a main dish (e.g., making a tuna salad or bean chili). Notably, consumption of protein-rich foods decreases appetite given the fact it takes longer for the body to break down the protein in these foods meaning protein might aid in controlling intake of excess kcalories. ***Social cognitive belief(s) targeted: Promoting Self-Efficacy, Improving Outcome Expectancy; Self-determination theory constructs potentially influenced: Competence.***

**Week Five**

***Physical Activity Tip:***

Again, social support is important to physical activity participation. If you are engaging in a new physical activity program, perhaps tell your family and close friends about your new program and related goals. Stating your plans/goals out loud not only increases the likelihood that you will continue this physical activity program, but your family and friends will surely ask about it at some point in the future meaning you may be held accountable for sticking to this program and achieving your health goals! ***Social cognitive belief(s) targeted: Enhancing Social Support; Self-determination theory constructs potentially influenced: Relatedness/Social Interactions.***

***Nutrition Tip:***

Foods like sour cream, cream, and regular cheese can be high in saturated and total fat. However, superb substitutes for the aforementioned foods are available in the form of low-fat yogurt, milk, and cheese, as well as soy alternatives—foods also often higher in protein content than their less healthy counterparts. Consider low-fat yogurt, milk, cheese, and soy alternatives to decrease saturated and total fat intake while also ensuring adequate intake of protein. ***Social cognitive belief(s) targeted: Promoting Self-Efficacy, Improving Outcome Expectancy; Self-determination theory constructs potentially influenced: Competence.***

**Week Six**

***Physical Activity Tip:***

One of the best ways to ensure you continue to participate in a physical activity program is finding an activity you enjoy. If you are an individual who prefers to work out alone, perhaps swimming, running, or biking suits you the best. For individuals who prefer to workout with others, consider group exercise classes such as yoga or step aerobics such as Zumba or dancing. Additionally, do not be afraid to mix and match different types of exercise! If you are going to sweat, you might as well be doing something that interests you! ***Social-cognitive belief(s) targeted: Increasing Enjoyment, Enhancing Social Support; Self-determination theory constructs potentially influenced: Self-Determination/Autonomy.***

***Nutrition Tip:***

Worried about a potluck or party derailing your goal of healthier eating? Keep in mind the following three tips. First, walk around the food table to see what options are available and decide upon what available foods are conducive to your nutritional goals and which are not. Second, ensure you create for yourself a colorful plate approximately half full of fruits and/or vegetables, with smaller portions of each food you choose. Eating foods of different colors will increase the variety of health-enhancing nutrients consumed while eating smaller portions of food will ensure you do not eat too much. Finally, consider water or unsweetened iced tea as drink options while at a potluck or party instead of sugar-sweetened and/or high-calorie options such as soda, juices, punch, or alcohol. In this way, you can still have fun with friends without worrying about derailing your health goals! ***Social cognitive belief(s) targeted: Promoting Self-Efficacy, Improving Outcome Expectancy, Decreasing Barriers; Self-determination theory constructs potentially influenced: Self-Determination/Autonomy, Competence.***

**Week Seven**

***Physical Activity Tip:***

Crank those tunes! If music is something you know will get you motivated to engage in your workout, consider investing in some athletic-oriented headphones that are sweat resistant and capable of staying in your ears during exercise. Keeping an up-to-date playlist of your favorite songs will allow you to have a better workout. This is especially true of exercisers preferring to exercise indoors. However, if exercising outdoors, consider leaving the headphones at home and enjoying the sounds and scenery as use of headphones while exercising outdoors can put you in danger. ***Social-cognitive belief(s) targeted: Increasing Enjoyment; Self-determination theory constructs potentially influenced: Competence.***

***Nutrition Tip:***

Coffee is a daily beverage for many. Yet, how an individual prepares their coffee can have a direct effect on their nutritional goals. Therefore, think about the following when getting your daily coffee. First, consider downsizing to a smaller drink, nixing whipped cream and caramel, and reducing or eliminating the addition of syrups to your drink. Second, if you like to add dairy to your coffee, look at adding low- or non-fat milk instead of whole fat milk. Not only will this substitution decrease caloric intake, but this substitution will also reduce saturated and total fat intake. Finally, when purchasing a muffin or pastry, perhaps consider splitting it with a friend as these foods can be higher in kcalories. ***Social cognitive belief(s) targeted: Promoting Self-Efficacy; Self-determination theory constructs potentially influenced: Competence.***

**Week Eight**

***Physical Activity Tip:***

Journaling is a great way to increase the likelihood of engagement in physical activity! Writing down not only your workouts, but the feelings associated with those workouts forces you to reflect upon different aspects of your exercise session. This reflection can have a profound impact on your motivation to continue your physical activity program and your desire to continue to experience the mental and physical benefits coming about as a result of participating in a regular physical activity routine. Try journaling today! ***Social-cognitive belief(s) targeted: Improving Outcome Expectancy, Improving Outcome Expectancy; Self-determination theory constructs potentially influenced: Competence.***

***Nutrition Tip:***

Limit eating out to once a week. When eating out, however, consider how you might improve the health of your order. For instance, you could look for entrees featuring vegetables such as a chicken or vegetable stir-fry. Further, you might consider ordering steamed options as steamed foods are often lower in saturated and total fat than fried foods. Finally, asking for additional oils or sauces on the side is a request many restaurants gladly accommodate of which will allow you more control of the caloric, salt, and fat content of your food. ***Social cognitive belief(s) targeted: Promoting Self-Efficacy, Decreasing Barriers; Self-determination theory constructs potentially influenced: Self-Determination/Autonomy, Competence.***

**Week Nine**

***Physical Activity Tip:***

Did you know that literature states that around 60 days is needed to make a new behavior a habit? Think about physical activity in the same manner. If you can engage in physical activity on a regular basis—say four times a week—over the course of two months, you will likely end up craving the good feeling physical activity provides when you miss a workout. Therefore, think about setting a start date and a follow-up date two months out. Then, plan to exercise a specified number of times per week over the next two months. At the follow-up date, reflect upon what your physical activity program means in the context of your daily routine. You will likely find that physical activity is something that you now crave and cannot do without! ***Social-cognitive belief(s) targeted: Promoting Self-Efficacy, Decreasing Barriers, Improving Outcome Expectancy; Self-determination theory constructs potentially influenced: Self-Determination/Autonomy, Competence.***

***Nutrition Tip:***

Fiber is a nutrient which aids in decreasing levels of low-density lipoprotein cholesterol (i.e., the “bad” cholesterol) and promoting increased high-density lipoprotein cholesterol (i.e., the “good” cholesterol) while also promoting better digestive functioning. One way to ensure adequate intake of fiber is to increase consumption of foods high in whole grains. Foods such as bread, rice, oats, and granola can be great sources of whole grains; however, not all of these foods are created equal. To ensure the food you are considering purchasing is high in whole grains, look for terms such as “100% whole grain” or “100% whole wheat” on the ingredient label. Not only will these foods provide higher fiber per serving, but they will also possess greater amounts of nutrients per serving compared to more refined grains. ***Social-cognitive belief(s) targeted: Promoting Self-Efficacy; Self-determination theory constructs potentially influenced: Competence.***

**Week Ten**

***Physical Activity Tip:***

Individuals new to a physical activity program often believe that they need to exercise every day to receive the benefits of exercise. However, this could not be further from the truth! Rest and recovery is a good thing. Consider exercising a maximum of 4 days a week if you are just beginning a physical activity program. Further, consider splitting these 4 days up over the course of the week to allow for your body to recover. For example, you could work out on Monday and Tuesday, rest on Wednesday, workout on Thursday and Friday, and take the weekend off. This rest will allow your body to experience the maximum amount of adaptations from your workouts! **Social-cognitive belief(s) targeted*: Promoting Self-Efficacy, Decreasing Barriers, Improving Outcome Expectancy; Self-determination theory constructs potentially influenced: Self-Determination/Autonomy, Competence.***

***Nutrition Tip:***

Got a “sweet tooth”?! If so, you are not the only one! The good news is, your sweet tooth does not have to adversely affect your pursuit of healthier eating behaviors. Indeed, consider making a fruit salad or yogurt parfait for a cold dessert or, for a delicious hot dessert option, baked apples with cinnamon as a topping is a great choice. What a great way to blend the enjoyment of satisfying a sweet tooth with engaging in more nutritious eating! **Social-cognitive belief(s) targeted*: Promoting Self-Efficacy, Increasing Enjoyment, Decreasing Barriers; Self-determination theory constructs potentially influenced: Competence***

**Week Eleven**

***Physical Activity Tip:***

Sleep may not seem important to physical activity, but it is. Not getting enough sleep is a sure-fire way to experience decreases in motivation for engaging in physical activity. Therefore, aim for 6 to 8 h of sleep each night. Moreover, try to cut out all screen time in the 10 to 15 min prior to going to sleep as watching TV or using your computer/smartphone to cruise social media or read the news decreases the body’s ability to produce melatonin, a key sleep hormone. Finally, consider removing any TV from the bedroom and/or not playing music while you sleep as this background noise can actually decrease sleep quality. Making these small changes can go a long way in helping you feel more rested and ready to engage in physical activity (and life) the next day! ***Social-cognitive belief(s) targeted: Promoting Self-Efficacy, Decreasing Barriers; Self-determination theory constructs potentially influenced: Competence.***

***Nutrition Tip:***

Look at your plate. What do you see? Ideally, you should see half your plate as including fruit and/or vegetables. Fruits and vegetables include nutrients vital to good health. Further, and more specifically, fruit can be a great natural substitute for candy or other dessert foods while many vegetables provide fiber important to the promotion of proper blood lipid levels (e.g., cholesterol). Notably, when eaten in proper amounts, fruits and vegetables are often lower in kcalories than other sides included with a meal. ***Social-cognitive belief(s) targeted: Promoting Self-Efficacy; Self-determination theory constructs potentially influenced: Competence.***

**Week Twelve**

***Physical Activity Tip:***

Face it, you have put in the time in the gym and, perhaps, even lost a little weight in the process. Consider a monthly or bi-monthly reward. This reward can be anything from the purchase of that one shirt that you have been dying to add to your wardrobe to a night out with your significant other. Yet, whatever the reward is, make sure that it does not derail your quest for better health and participation in physical activity. ***Social-cognitive belief(s) targeted: Improving Outcome Expectancy; Self-determination theory constructs potentially influenced: Self-Determination/Autonomy.***

***Nutrition Tip:***

Healthy eating is always more fun with friends! Consider a “Healthy Cooking Night” with friends where each individual is asked to search for a healthy entrée, side, dessert or beverage to cook/bake. Once each individual has found a recipe for their aspect of the meal, spend the night together cooking and baking! Not only will this increase the likelihood of engagement in healthy eating behaviors, but it is promoting support between each individual for participation in healthier lifestyle behaviors! ***Social-cognitive belief(s) targeted: Promoting Self-Efficacy, Increasing Enjoyment, Promoting Social Support; Self-determination theory constructs potentially influenced: Relatedness/Social Interactions.***

## References

[1] Deliens T., Deforche B., De Bourdeaudhuij I., Clarys P. (2015). Determinants of physical activity and sedentary behaviour in university students: A qualitative study using focus group discussions. BMC Public Health.

[2] Desai M., Miller W., Staples B., Bravender T. (2008). Risk Factors Associated With Overweight and Obesity in College Students. J. Am. Coll. Health.

[3] Peterson N., Sirard J., Kulbok P., DeBoer M., Erickson J. (2018). Sedentary behavior and physical activity of young adult university students. Res. Nurs. Health.

[4] Larson N., Neumark-Sztainer D., Story M., Burgess-Champoux T. (2010). Whole-Grain Intake Correlates among Adolescents and Young Adults: Findings from Project EAT. J. Am. Diet. Assoc..

[5] Racette S., Deusinger S., Strube M., Highstein G., Deusinger R. (2005). Weight changes, exercise, and dietary patterns during freshman and sophmore years of college. J. Am. Coll. Health.

[6] Giskes K., van Lenthe F., Avendano-Pabon M., Brug J. (2011). A systematic review of environmental factors and obesogenic dietary intakes among adults: Are we getting closer to understanding obesogenic environments?. Obes. Rev..

[7] Malhotra R., Østbye T., Riley C., Finkelstein E. (2013). Young adult weight trajectories through midlife by body mass category. Obesity.

[8] Cawley J., Meyerhoefer C. (2012). The medical care costs of obesity: An instrumental variables approach. J. Health Econ..

[9] Welch V., Petkovic J., Simeon R., Presseau J., Gagnon D., Hossain A., Pardo Pardo J., Pottie K., Rader T., Sokolovski A. (2018). Interactive social media interventions for health behaviour change, health outcomes, and health equity in the adult population (protocol). Cochrane Database Syst. Rev..

[10] Partridge S., Redfern J. (2018). Obesity prevention in young people: The role of technology in primary care. J. Prim. Care Gen. Pract..

[11] Henriksen A., Mikalsen M., Woldaregay A., Muzny M., Hartvigsen G., Sanders J., Wark P., Winfree K., Fallahzadeh R., Fernández C. (2018). Using Fitness Trackers and Smartwatches to Measure Physical Activity in Research: Analysis of Consumer Wrist-Worn Wearables. J. Med. Internet Res..

[12] Muller A., Maher C., Vandelanotte C., Hingle M., Middelweerd A., Lopez M.L., Desmet A., Short C.E., Bardus M., Hand R. (2018). Physical Activity, Sedentary Behavior, and Diet-Related eHealth and mHealth Research: Bibliometric Analysis. J. Med. Internet Res..

[13] Schembre S., Liao Y., Robertson M., Dunton G.F., Kerr J., E Haffey M., Burnett T., Basen-Engquist K., Turner-McGrievy B., Gomez I.N. (2018). Just-in-Time Feedback in Diet and Physical Activity Interventions: Systematic Review and Practical Design Framework. J. Med. Internet Res..

[14] Zeng N., Gao Z., Gao Z. (2017). Health Wearable Devices and Physical Activity Promotion. Technology in Physical Activity and Health Promotion.

[15] Kim Y., Lumpkin A., Lochbaum M., Stegemeier S., Kitten K. (2018). Promoting physical activity using wearable activity tracker in college students: A cluster randomized trial. J. Sport Sci..

[16] Melton B., Buman M., Vogel R., Harris B., Bigham L. (2016). Wearable devices to improve physical activity and sleep: A randomized controlled trial of college-aged African American women. J. Black Stud..

[17] Thorndike A., Mills S., Sonnenberg L., Palakshappa D., Gao T., Pau C.T., Regan S. (2014). Activity Monitor Intervention to Promote Physical Activity of Physicians-In-Training: Randomized Controlled Trial. PLoS ONE.

[18] Wang J., Cadmus-Bertram L., Natarajan L., White M.M., Madanat H., Nichols J.F., Ayala G.X., Pierce J.P. (2015). Wearable sensor/device (fitbit one) and SMS text-messaging prompts to increase physical activity in overweight and obese adults: A randomized controlled trial. Telemed. E-Health.

[19] Thompson W., Kuhle C., Koepp G., McCrady-Spitzer S., Levine J. (2014). “Go4Life” exercise counseling, accelerometer feedback, and activity levels in older people. Arch. Gerontol. Geriatr..

[20] Cadmus-Bertram L., Marcus B., Patterson R., Parker B., Morey B. (2015). Randomized Trial of a Fitbit-Based Physical Activity Intervention for Women. Am. J. Prev. Med..

[21] Elliot C., Hamlin M. (2018). Combined diet and physical activity is better than diet or physical activity alone at improving health outcomes for patients in New Zealand’s primary care intervention. BMC Public Health.

[22] Pew Research Center (2017). Social Media Fact Sheet. http://www.pewinternet.org/fact-sheet/social-media/.

[23] Cavallo D., Tate D., Ries A., Brown J., DeVellis R., Ammerman A. (2012). A social-media based physical activity intervention: A randomized controlled trial. Am. J. Prev. Med..

[24] Bunn J., Navalta J., Fountaine C., Reece J. (2018). Current State of Commercial Wearable Technology in Physical Activity Monitoring 2015–2017. Int. J. Exerc. Sci..

[25] National Wellness Institute (2018). The Six Dimensions of Wellness. http://www.nationalwellness.org/?page=Six_Dimensions.

[26] Kohl H., Murray T., Kohl H., Murray T. (2012). Program and policy evaluation for physical activity and public health. Foundations of Physical Activity and Public Health.

[27] Matthews C., Hagströmer M., Pober D., Bowles H. (2012). Best practices for using physical activity monitors in population-based research. Med. Sci. Sports Exerc..

[28] Brug J., Oenema A., Ferreira I. (2005). Theory, evidence, and intervention mapping to improve behavior, nutrition, and physical activity interventions. Int. J. Behav. Nutr. Phys..

[29] Patten M., Patten M. (2014). The role of theory in research. Understanding Research Methods: An overview of the Essentials.

[30] Bandura A. (2004). Health Promotion by Social Cognitive Means. Health Educ. Behav..

[31] Sriramatr S., Silalertdetkul S., Wachirathanin P. (2016). Social cognitive theory associated with physical activity in undergraduate students: A cross-sectional study. Pac. Rim Int. J. Nurs. Res..

[32] Mirzaei-Alavijeh M., Soroush A., Nasirzadeh M., Hatamzadeh N., Zinat-Motlagh F., Jalilian F., Mohammadi M., Mahboubi M. (2018). Socio-Cognitive Determinants of Regular Physical Activity among College Students. World Fam. Med..

[33] Marr J., Wilcox S. (2015). Self-efficacy and Social Support Mediate the Relationship Between Internal Health Locus of Control and Health Behaviors in College Students. Am. J. Health Educ..

[34] Farmanbar R., Niknami S., Lubans D., Hidarnia A. (2013). Predicting exercise behaviour in Iranian college students: Utility of an integrated model of health behaviour based on the transtheoretical model and self-determination theory. Health Educ. J..

[35] Phillips L., Chamberland P.-E., Hekler E., Abrams J., Eisenberg M. (2016). Intrinsic rewards predict exercise via behavioral intentions for initiators but via habit strength for maintainers. Sport Exerc. Perform..

[36] National Center of Complementary and Integrative Health (2017). Pilot Studies: Common Uses and Misuses. https://nccih.nih.gov/grants/whatnccihfunds/pilot_studies.

[37] Leon A., Davis L., Kraemer H. (2011). The role and interpretation of pilot studies in clinical research. J. Psychiatr. Res..

[38] Schulz K., Altman D., Moher D. (2010). CONSORT 2010 Statement: Updated guidelines for reporting parallel group randomised trials. BMC Med..

[39] U.S. Department of Health and Human Services (2018). Physical Activity Guidelines for Americans.

[40] U.S. Department of Agriculture (2015). Scientific Report of the 2015 Dietary Guidelines Advisory Committee. http://health.gov/dietaryguidelines/2015-scientific-report/pdfs/scientific-report-of-the-2015-dietary-guidelines-advisory-committee.pdf.

[41] Thompson F., Subar A., Smith A., Midthune D., Radimer K.L., Kahle L.L., Kipnis V. (2002). Fruit and vegetable assessment: Performance of 2 new short instruments and a food frequency questionnaire. J. Am. Diet. Assoc..

[42] World Medical Association (2018). World Medical Association Declaration of Helsinki: Ethical Principles for Medical Research Involving Human Subjects. https://www.wma.net/policies-post/wma-declaration-of-helsinki-ethical-principles-for-medical-research-involving-human-subjects/.

[43] Ayabe M., Junichiro A., Kumahara H., Tanaka H. (2011). Assessment of minute-by-minute stepping rate of physical activity under free-living conditions in female adults. Gait Posture.

[44] Tudor-Locke C., Camhi S., Leonardi C., Johnson W.D., Katzmarzyk P.T., Earnest C.P., Church T.S. (2011). Patterns of adult stepping cadence in the 2005–2006 NHANES. Prev. Med..

[45] Herrmann S., Barreira T., Kang M., Ainsworth B. (2013). How many hours are enough? Accelerometer wear time may provide bias in daily activity estimates. J. Phys. Act. Health.

[46] Trost S., McIver K., Pate R. (2005). Conducting accelerometer-based activity assessments in field-based research. Med. Sci. Sport Exerc..

[47] Golding L., Meyers C., Sinning W. (1998). Y’s Way to Physical Fitness: The Complete Guide to Fitness Testing and Instruction.

[48] Aandstad A., Holtberget K., Hageberg R., Holme I., Anderssen S. (2014). Validity and Reliability of Bioelectrical Impedance Analysis and Skinfold Thickness in Predicting Body Fat in Military Personnel. Mil. Med..

[49] Carlson J., Sallis J., Wagner N., Calfas K.J., Patrick K., Groesz L.M., Norman G.J. (2012). Brief physical activity-related psychosocial measures: Reliability and construct validity. J. Phys. Act. Health.

[50] Ommundsen Y., Page A., Po-Wen K., Cooper A. (2008). Cross-cultural, age and gender validation of a computerised questionnaire measuring personal, social and environmental associations with children’s physical activity: The European Youth Heart Study. Int. J. Behav. Nutr. Phys..

[51] Trost S., Pate R., Saunders R., Ward D., Dowda M., Felton G. (1997). A Prospective Study of the Determinants of Physical Activity in Rural Fifth-Grade Children. Prev. Med..

[52] Sechrist K., Walker S., Pender N. (1987). Development and psychometric evaluation of the exercise benefits/barriers scale. Res. Nurs. Health.

[53] Gesell S., Reynolds E., Ip E., Fenlason L.C., Pont S.J., Poe E.K., Barkin S.L. (2008). Social influences of self-reported physical activity in Latino children. Clin. Pediatr..

[54] Saunders R., Pate R., Felton G., Dowda M., Weinrich M.C., Ward D.S., Parsons M.A., Baranowski T. (1997). Development of Questionnaires to Measure Psychosocial Influences on Children’s Physical Activity. Prev. Med..

[55] Tavakol M., Dennick R. (2011). Making sense of Cronbach’s alpha. Int. J. Med. Educ..

[56] McAuley E., Duncan T., Tammen V. (1987). Psychometric properties of the intrinsic motivation inventory in a competitive sport setting: A confirmatory factor analysis. Res. Q. Exerc. Sport.

[57] Kipnis V., Subar A., Midthune D., Freedman L.S., Ballard-Barbash R., Troiano R.P., Bingham S., Schoeller D.A., Schatzkin A., Carroll R.J. (2003). Structure of dietary measurement error: Results of the OPEN biomarker study. Am. J. Epidemiol..

[58] Moshfegh A., Rhodes D., Baer D., Murayi T., Clemens J.C., Rumpler W.V., Paul D.R., Sebastian R.S., Kuczynski K.J., A Ingwersen L. (2008). The US Department of Agriculture Automated Multiple-Pass Method reduces bias in the collection of energy intakes. Am. J. Clin. Nutr..

[59] Baranowski T., Willett W. (2013). 24-hour recall and diet record methods. Nutritional Epidemiology.

[60] Willett W., Willet W. (2013). Nature of variation in diet. Nutritional Epidemiology.

[61] Wang Y., Bleich S., Gortmaker S. (2008). Increasing Caloric Contribution From Sugar-Sweetened Beverages and 100% Fruit Juices Among US Children and Adolescents, 1988–2004. Pediatrics.

[62] Popkin B., E Armstrong L., Bray G., Caballero B., Frei B., Willett W. (2006). A new proposed guidance system for beverage consumption in the United States. Am. J. Clin. Nutr..

[63] Arem H., Moore S., Patel A., Hartge P., De Gonzalez A.B., Visvanathan K., Campbell P.T., Freedman M., Weiderpass E., Adami H.O. (2015). Leisure Time Physical Activity and Mortality: A Detailed Pooled Analysis of the Dose-Response Relationship. JAMA Intern. Med..

[64] Smetaniuk T., Johnson D., Creurer J., Block K., Schlegel M., Butcher S., Oosman S.N. (2017). Physial activity and sedentary behaviour of master of physical therapy students: An exploratory study of facilitators and barriers. Physiother. Can..

[65] Bopp M., Bopp C., Schuchert M. (2013). Active transportation to and on campus is associated with objectively measured fitness outcomes among college students. J. Phys. Act. Health.

[66] Sisson S., Tudor-Locke C. (2008). Comparison of cyclists’ and motorists’ utilitarian physical activity at an urban university. Prev. Med..

[67] Ekelund U., Steene-Johannessen J., Brown W., Fagerland M.W., Owen N., Powell K.E., Bauman A., Lee I.-M., Lancet Physical Activity Series 2 Executive Committee, Lancet Sedentary Behaviour Working Group (2016). Does physical activity attenuate, or even eliminate, the detrimental association of sitting time with mortality? A harmonised meta-analysis of data from more than 1 million men and women. Lancet.

[68] De Vos P., Hanck C., Neisingh M., Prak D., Groen H., Faas M. (2015). Weight gain in freshman college students and perceived health. Prev. Med. Rep..

[69] Li Y.-C., Li C.-I., Lin W.-Y., Liu C.-S., Hsu H.-S., Lee C.-C., Chen F.-N., Li T.-C., Lin C.-C. (2013). Percentage of Body Fat Assessment Using Bioelectrical Impedance Analysis and Dual-Energy X-ray Absorptiometry in a Weight Loss Program for Obese or Overweight Chinese Adults. PLoS ONE.

[70] Kenney W., Wilmore J., Costill D., Kenney W., Wilmore J., Costill D. (2015). Body composition and nutrition for sport. Physiology of Sport and Exercise.

[71] Kenney W., Wilmore J., Costill D., Kenney W., Wilmore J., Costill D. (2015). Adaptations to aerobic and anaerobic training. Physiology of Sport and Exercise.

[72] Von A.D., Ebert S., Ngamvitroj A., Park N., Kang D.-H. (2004). Predictors of health behaviors in college students. J. Adv. Nurs..

[73] Voorhees C. (2003). Personal, social, and physical environmental correlates of physical activity levels in urban Latinas. Am. J. Prev. Med..

[74] Farren G., Zhang T., Martin S., Thomas K. (2017). Factors related to metting physical activity guidelines in active college students: A social cognitive perspective. J. Am. Coll. Health.

[75] Blake H., Stanulewicz N., McGill F. (2017). Predictors of physical activity and barriers to exercise in nursing and medical students. J. Adv. Nurs..

[76] Grubbs L., Carter J. (2002). The Relationship of Perceived Benefits and Barriers to Reported Exercise Behaviors in College Undergraduates. Fam. Community Health.

[77] Kohl H., Murray T., Kohl H., Murray T. (2012). Overweight and obesity. Foundations of Physical Activity and Public Health.

[78] Poddar K., Hosig K., Anderson E., Nickols-Richardson S., Duncan S. (2010). Web-Based Nutrition Education Intervention Improves Self-Efficacy and Self-Regulation Related to Increased Dairy Intake in College Students. J. Am. Acad. Nutr. Diet..

