# Supplementary figures and images for: Use of Wearable Technology and Social Media to Improve Physical Activity and Dietary Behaviors among College Students: A 12-Week Randomized Pilot Study
Source: Int J Environ Res Public Health. 2019 Sep 25;16(19):3579. doi: 10.3390/ijerph16193579 (PMC6801802; doi:10.3390/ijerph16193579)

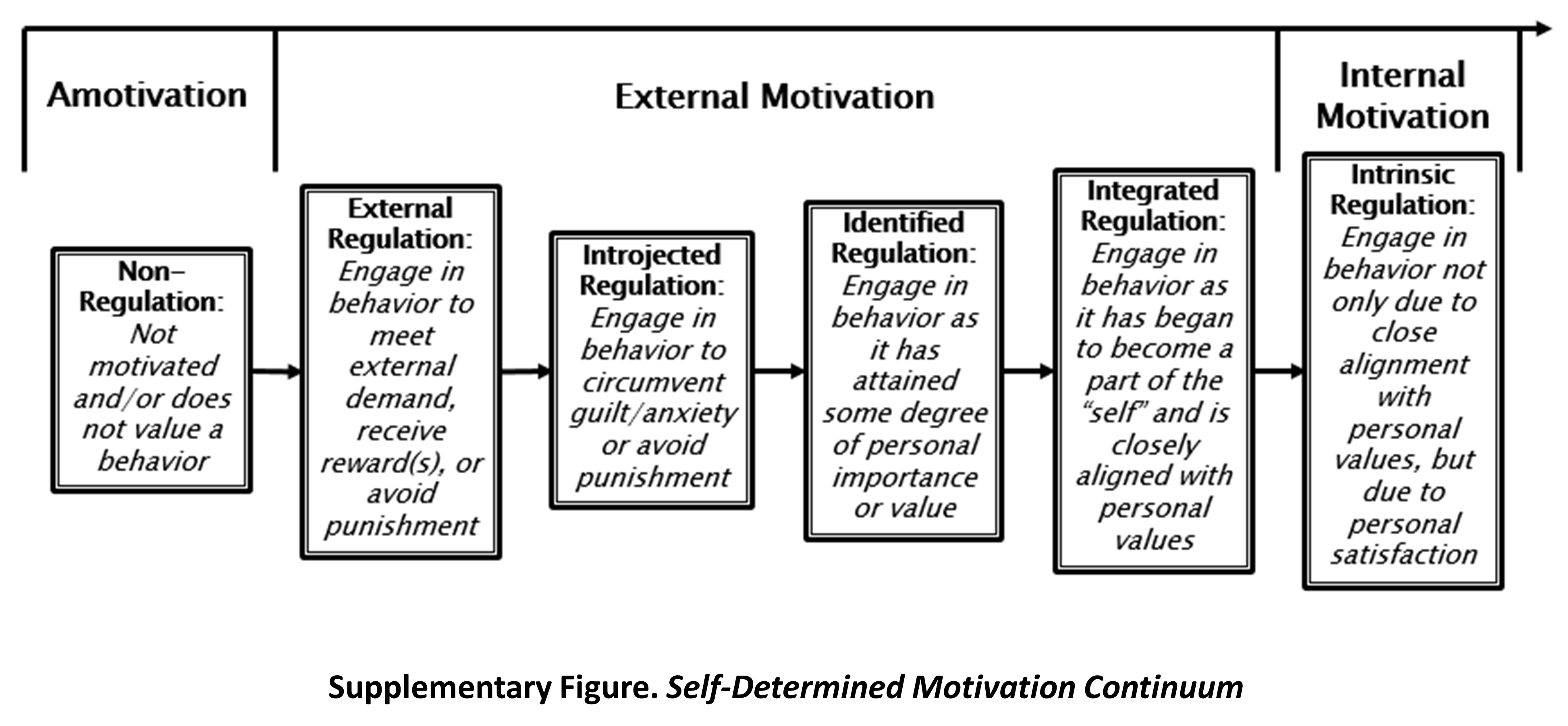

Supplement: Supplementary file 1 [file ijerph-16-03579-s001.zip › Fig S1-Self-Determined Motivation Continuum 09022019.tiff]
